# Microbial metabolites indole derivatives sensitize mice to D-GalN/LPS induced-acute liver failure *via* the Tlr2/NF-κB pathway

**DOI:** 10.3389/fmicb.2022.1103998

**Published:** 2023-01-06

**Authors:** Ziyuan Zhou, Baohong Wang, Xiaxia Pan, Jiawen Lv, Zhuoqi Lou, Yuqiu Han, Yuanyuan Yao, Jun Chen, Qiangqiang Wang, Lanjuan Li

**Affiliations:** State Key Laboratory for Diagnosis and Treatment of Infectious Diseases, National Clinical Research Center for Infectious Diseases, Collaborative Innovation Center for Diagnosis and Treatment of Infectious Diseases, The First Affiliated Hospital, Zhejiang University School of Medicine, Hangzhou, China

**Keywords:** microbial metabolites, indole derivatives, acute liver failure, toll-like receptor 2, NF-κB, gut microbiota

## Abstract

**Introduction:**

Acute liver failure (ALF) is a clinical condition with many causes, fast progression, and a poor prognosis. Previous research has indicated that microbial factors have a role in ALF, but a clear picture has yet to emerge.

**Methods:**

To investigate the specific involvement of microbial metabolites in ALF development, we pretreated D-GalN/LPS-induced ALF mice with indole derivatives, an influential class of gut microbial metabolites.

**Results:**

Contrary to their typical role as anti-inflammatory agents in the host, indole-3-acetic acid (IAA), indole-3-lactic acid (ILA), and indolepropionic acid (IPA) gavage sensitize mice to D-GalN/LPS-induced-ALF with a rapid rise in serum transaminases and histologic lesion. For a clearer picture, we performed comprehensive analysis for the IAA therapy. IAA markedly amplified inflammatory response and cellular damage. The transcriptome analysis indicated the participation of the TNF-α/NF-κB signaling pathway. The structure of gut microbiota in ileum and the expression of Toll-like receptor 2 (Tlr2) in the liver were also significantly changed.

**Discussion:**

In conclusion, IAA pretreatment can exacerbate D-GalN/LPS-induced ALF via probable Tlr2/NF-κB pathway involvement and ileac dysbiosis characterized by enriched gram-positive genus with potential pathogenesis. Microbial metabolites IAA may aggravate individual susceptibility to D-GalN/LPS-induced ALF. Further investigation of the underlying mechanism is needed, and intervention with indole derivatives and related commensal species should be undertaken with caution.

## Introduction

Acute liver failure (ALF) is a uncommon but lethal threat in clinical conditions (Stravitz and Lee, 2019). Various pathogenic causes such as drugs, viruses and alcohol can induce an abrupt liver dysfunction characterized by severe hepatic necrosis. Although the fatality rate is gradually decreased due to the improvement of the supportive treatment, the underlying pathogenic factors influencing symptom severity and individual sensitivity are still not thoroughly understood (Wendon et al., 2017). The fulminant onset and rapid progression limit further investigation, leaving ALF as a serious liver disease to be solved.

The gut-liver axis has been recognized as an influential factor in different liver diseases, which indicates the significance of intestinal microecology in liver diseases (Tripathi et al., 2018; Albillos et al., 2020; Chen et al., 2021). Different studies have reported the involvement of microbial factors in ALF development (Wu et al., 2022). On the one hand, microbial intervention with probiotic/prebiotic and FMT have exhibited therapeutic potential in different studies (Wang Q. et al., 2019; Liu Y. et al., 2021; Wang J. et al., 2022; Xia et al., 2022). It is helpful in the regulation of inflammation response. On the other hand, several reports revealed that gut bacteria serve as a pathogenic factor and the depletion of gut bacteria improves liver injury (Possamai et al., 2015; Gong et al., 2021; Zheng et al., 2022). These controversial findings suggest a complex role of gut microecology in ALF progression and urge further investigation.

Many commensal species are capable of metabolizing dietary tryptophan (Trp) into an important kind of microbial metabolites, indole derivatives which mainly exhibit anti-inflammatory function in liver diseases (Ji et al., 2019; Zhao et al., 2019; Ma et al., 2020). To further explore the role of microbial factor in ALF, we decide to examine the effect of several indole derivatives including IAA, ILA, and IPA, which have been widely investigated in different liver diseases (Qayed et al., 2021; Wang B. et al., 2022). However, our study indicates that indole derivatives, especially IAA pretreatment, can significantly sensitize mice to D-GalN/LPS induced-ALF, contradicting previous expectations. Further analysis revealed that IAA can induce dysbiosis in ileum characterized by increased gram-positive (G+) pathogen and decreased beneficial species, which may be responsible for the up-regulation of liver Tlr2/NF-κB pathway under D-GalN/LPS challenge. Our findings suggest that microbial metabolites, indole derivatives, in particular IAA, may act as a pathogen factor and aggravate individual susceptibility in ALF. Thus, the associated microbial interventions need further validation.

## Materials and methods

### Chemicals

D-Gal (G0500) was purchased from Sigma Aldrich (St Louis, MO, United States). LPS from Escherichia coli O55:B5 (#L2880) was purchased from Sigma Aldrich (St Louis, MO, United States). FICZ (#SML1489) was purchased from Sigma Aldrich (St Louis, MO, United States). Indole-3-lactic acid (#I157602) was purchased from Aladdin (Shanghai, China). Indole-3-acetic acid (#I101074) was purchased from Aladdin (Shanghai, China). 3-Indolepropionic acid (#I103959) was purchased from Aladdin (Shanghai, China).

### Animals and treatment

Hangzhou Ziyuan Experimental Animal Co., Ltd. supplied us with C57BL/6 mice. All of the animals were housed in specific pathogen-free (SPF) conditions at the animal facility of the First Affiliated Hospital of Zhejiang University Medical School. All the animals were grown at a temperature of 22 ± 2°C with a light/dark cycle of 12 h, and fed with standard chow diet. All 60 male 6-to 8-week-old wild-type mice (all on C57BL/6 background and weight from 20 to 22 g) were randomly divided into 6 groups: (1) IAA + D-GalN/Lps (IAAL) group, (2) ILA + D-GalN/Lps (ILAL) group, (3) IPA + D-GalN/Lps (IPAL) group, (4) Ficz +D-GalN/Lps (FiAL) group, (5) Vehicle+D-GalN/Lps (VeAL) group, and (6) Vehicle (Veh) group. IAAL was administered orally in the form of 5 mg/ml IAA with 200 μl volume per mouse daily for 1 week. The IAA stock solution (25 mg/ml, 5) was produced in sterile saline and dissolved by giving NaOH (1 N) and adjusting the pH to 7.2–7.4 with 25% (v/v) HCl. The gavage solution was diluted to a final concentration of 5 mg/ml using sterile saline. ILAL and IPAL were orally administrated with 2.5 mg/ml of ILA and 7.5 mg/ml of IPA with 200 μl volume per mouse per day for 1 week, respectively. ILA stock solution (12.5 mg/ml, 5×) and IPA stock solution (32.5 mg/ml, 5×) were prepared and dissolved in the same way as IAA. The final concentrations of ILA and IPA were 2.5 mg/ml and 7.5 mg/ml, respectively. The dosage of indole derivatives and the method to dissolve was based on the previous studies (Ji et al., 2019; Xiao et al., 2020) and our preliminary study which exhibited good safety and tolerance for mice. FiAL was orally administrated with 5 μg/1 ml of FICZ dissolved in sterile saline, 200 μl per mouse in day 2 and 6. The ALF model was created on day 8 through intraperitoneal injection of 400 mg/kg D-Gal and 50 μg/kg LPS dissolved in sterile saline (10 μl/g total injection volume). On day 8, Veh was administered intraperitoneally with sterile saline (final injection volume: 10 μl/g). The vehicle therapy consisted of daily oral administration of 200 μl of sterile saline. The mice were fasted overnight and sacrificed 6 h after intraperitoneal injection of D-Gal/LPS. The liver and feces were promptly frozen in liquid nitrogen and stored in a refrigerator at −80°C for future use.

The animal and treatment procedures were consistent with the National Institutes of Health’s standard for the care and use of laboratory animals and were approved by the Animal Care Ethics Committee of The First Affiliated Hospital, College of Medicine, Zhejiang University, China.

### Biochemical parameters analysis and histopathological examination

Serum was collected from the supernatant of the whole blood samples by centrifugation at 3,000 rpm at 4°C for 15 min. Serum was isolated and stored in −80°C refrigerator. Serum ALT and AST transaminases were determined using BS-220 Chemistry Analyzer (Mindray, Shengzhen, China). Hepatic tissue was removed, rinsed in ice-cold phosphate-buffered saline (PBS), and then preserved for 24 h in 4% paraformaldehyde. The 5 m-thick tissue slices were produced for hematoxylin and eosin (H&E) staining, while the remaining sections were embedded in paraffin. Samples were scanned and visualized by an 3DHISTECH Pannoramic 250 FLASH and caseviewer 2.4.^1^ The degree of liver histologic injury was measured according to the modified HAI scores (Ishak et al., 1995; Desmet, 2003). Each sample was examined at 40× for five random visual fields.

### TUNEL staining assay

Terminal deoxynucleotidyl transferase-mediated deoxyuridine triphosphate nick-end labeling (TUNEL) analysis was conducted on paraffin-embedded (5 μm) mouse liver slices treated with Leica AR9640 following antigen retrieval. BrightGreen Apoptosis Detection Kit (Vazyme, Nanjing, China) was used to perform TUNEL staining in accordance with the manufacturer’s instructions. Nucleus was labeled with DAPI. All images were scanned and visualized by 3DHISTECH Pannoramic 250 FLASH and caseviewer 2.4. Each sample was examined at 40× for five random visual fields.

### Quantitative real-time polymerase chain reaction (RT-qPCR)

Hepatic tissues’ total RNA was extracted with a Qiagen RNeasy Plus Mini Kit (QIAGEN, Hilden, Germany) and measured by a NanoDrop 2000 spectrophotometer (Thermo Fisher, MA, United States). Following the manufacturer’s instructions, 500 ng of RNA was reverse-transcribed to cDNA using PrimeScript RT Master Mix (Perfect Real Time) (TAKARA, Beijing, China). Quantitative polymerase chain reaction (qPCR) was done using a TB Green® Premix Ex TaqTM (Tli RNaseH Plus) (TAKARA, Beijing, China) kit including forward and reverse primers and cDNA template (100 ng) in an Applied Biosystems VIIA7 instrument (Thermo Fisher Scientific). The amplification protocol was as follows: 95°C for 30 s, 40 cycles of 95°C for 5 s, 60°C for 30 s, and dissociation step. The relative mRNA expression of target genes was standardized utilizing GAPDH as the reference gene and computed using the comparative CT technique. The sequences of RT-qPCR primers are included in Supplementary Table S1 of the Supplementary material.

### Cytokine bead array

Serum cytokines were quantified with the LEGENDplex^™^ Mouse Inflammation Panel (13-plex) with Filter Plate (Biolegend, CA, United States) according to the protocol provided by the manufacturer with flow cytometry method.

### Transcriptive activity of NF-κB (P65) assay

Nuclear extract was extracted with Cryopreserved sample nucleoprotein Extraction Kit (Bio-lab, Beijing, China). Protein concentration was measured by Enhanced BCA Protein Assay Kit (Beyotime, Shanghai, China).

The transcriptive activity of NF-κB (P65) was assessed by NFkB p65 Transcription Factor Assay Kit (Abcam, MA, United States) with nuclear extract. All the experiments were performed according to manufacturer’s protocol.

### Transcriptome analysis

mRNA molecules were extracted from whole RNA using magnetic beads to which oligo (dT) was coupled. mRNA molecules were fragmented into little fragments using a fragmentation reagent after a certain length of reaction at the correct temperature. Using random hexamer primed reverse transcription, first strand cDNA was synthesized, followed by second strand cDNA synthesis. The end of the generated cDNA was repaired, and then it was 3′ adenylated. These 3′ adenylated cDNA segments were ligated using adapters at their ends. cDNA fragments containing adapters from the previous stage were amplified using a PCR technique. Ampure XP Beads (AGENCOURT) were used to purify PCR products, which were then dissolved in EB solution. On the 2,100 bioanalyzer from Agilent Technologies, the library was verified. The double-stranded PCR products were denatured by heat and then circularized using the splint oligo sequence. As the final library, the single-strand circular DNA (ssCir DNA) was prepared. The library was amplified using phi29 to create DNA nanoballs (DNB) containing over 300 copies of a single molecule. The DNBs were loaded onto the patterned nanoarray, and pair-end 150-base readings were created *via* combinatorial Probe Anchor Synthesis (cPAS).

### Bacteria 16S RNA

Using the CTAB/SDS technique, complete genome DNA was extracted from samples. On 1% agarose gels, DNA concentration and purity were evaluated. Using sterile water, DNA was diluted to 1 ng/l based on its concentration. Using particular primers and barcodes, 16S rRNA genes in various locations (16S V3-V4) were amplified. All PCR mixtures comprised 15 l of Phusion^®^ High-Fidelity PCR Master Mix (New England Biolabs), 0.2 M of each primer, and 10 ng of target DNA, and cycling conditions included a 1 min initial denaturation at 98°C, 30 cycles at 98°C (10 s), 50°C (30 s), and 72°C (30 s), and a 5 min extension at 72°C. For DNA detection, mix PCR results with an equivalent amount of 1× loading buffer (including SYB green) and run electrophoresis on a 2% agarose gel. The PCR products were combined in identical amounts, and then they were purified using a Qiagen Gel Extraction Kit (Qiagen, Germany). Following the manufacturer’s instructions, NEBNext^®^ UltraTM IIDNA Library Prep Kit was used to construct sequencing libraries (Cat No. E7645). On the Qubit@ 2.0 Fluorometer (Thermo Scientific) and Agilent Bioanalyzer 2,100 system, the library’s quality was assessed. The library was sequenced on an Illumina NovaSeq device to yield 250 bp paired-end reads.

### Statistical analysis

Data are presented as mean ± SEM. Statistical analysis of non-sequence data between two or multiple groups was performed on GraphPad Prism 9 (CA, United States). Student’s t-test, and one-way analysis of variance (ANOVA) which is followed by Tukey’s or uncorrected Fisher’s LSD multiple comparisons test were used to examine the statistic difference. *P* values were considered statistically significant at <0.05. The statistical analysis of transcriptome sequence and 16S RNA are listed in the Supplementary material.

## Results

### Indole derivatives (IAA, ILA, IPA) sensitize mice to D-GalN/LPS induced-liver injury

Previous studies have highlighted the anti-inflammation and hepatoprotective role of the microbial metabolite indole derivatives in different liver diseases (Ji et al., 2019; Zhao et al., 2019). To investigate the effect of indole derivatives on D-GalN/LPS induced-acute liver injury (ALI), wild-type mice are pretreated with indole derivatives (IAA, ILA, IPA) or vehicle saline for a week, the acute liver injury (ALI) model is established on day eight with D-GalN/LPS injection. 4–5 h after D-GalN/LPS injection, some mice have begun to exhibit an adynamic condition characterized by reduced activity. Unexpectedly, mice pretreated with indole derivatives appeared to exhibit more severe symptoms and left less circulating blood and colon contents for sampling on the sixth hour, contradicting our previous expectations.

**Figure 1:**
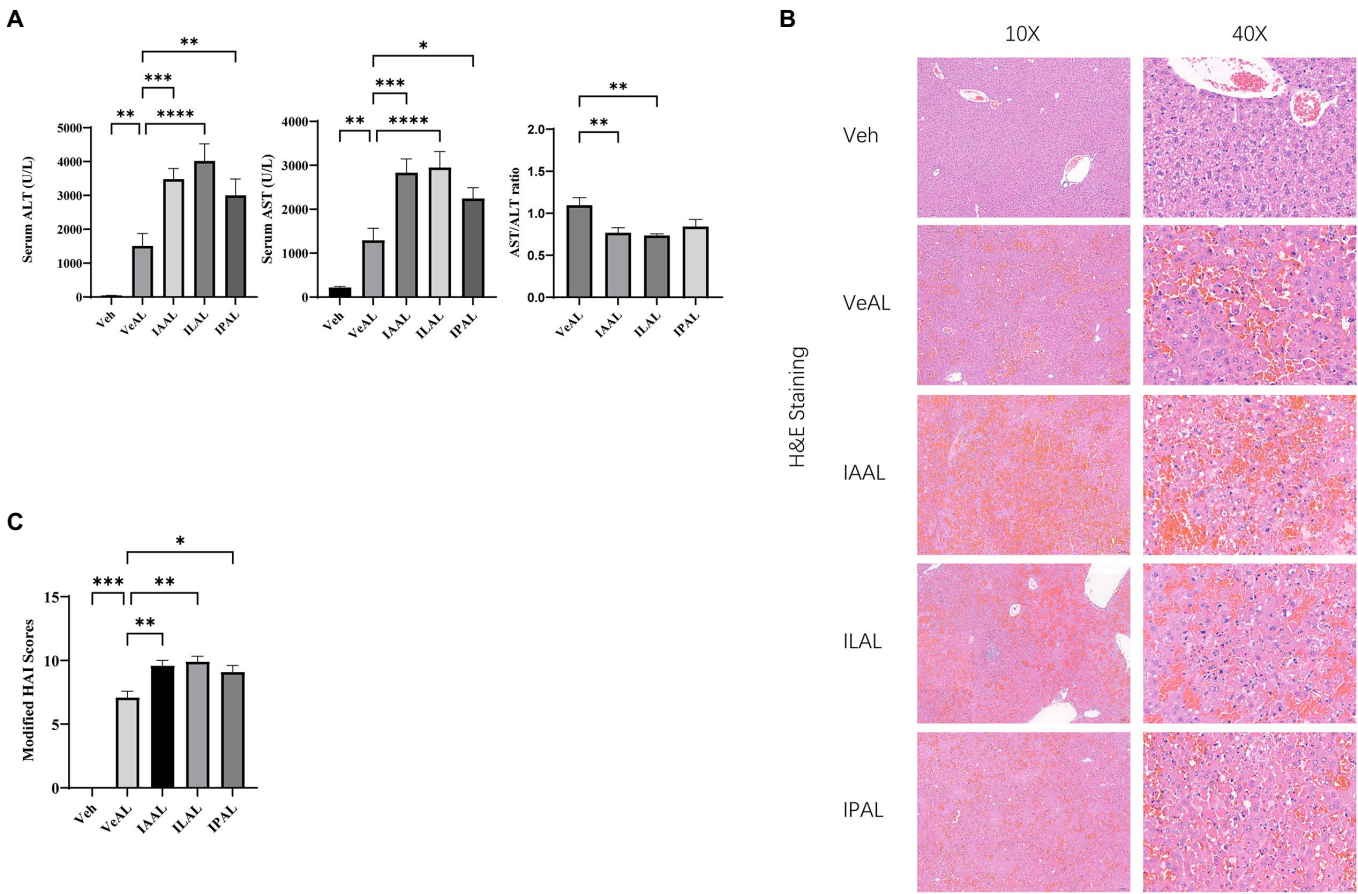
Pretreatment of indole derivatives sensitize mice to D-GalN/LPS induced-liver injury. **(A)** Serum levels of ALT and AST in all groups, and AST/ALT ratios in four ALF groups. **(B)** Representative liver H&E staining (original magnification × 10/40, scale bar = 100 μm/20 μm). **(C)** Liver pathological modified HAI. The data are presented as the mean ± SEM. *p < 0.05; **p < 0.01; ***p < 0.001 and ****p < 0.0001 for the comparison. Veh, Vehicle (saline control); VeAL, Vehicle + D-GalN/LPS; IAAL, IAA + D-GalN/LPS; ILAL, ILA + D-GalN/LPS; IPAL, IPA + D-GalN/LPS. *n* = 10 in each group.

To check the severity of ALI in each group. We evaluate the alanine aminotransferase (ALT) and aspartate aminotransferase (AST) levels as typical markers of liver injury. Serum ALT and AST levels increased significantly with D-GalN/LPS injection. The pretreatment with indole derivatives (IAA, ILA, IPA) aggravates the acute injury in liver with elevated aminotransferase and decreased AST/ALT ratios (Figure 1A). We then evaluated the histologic injury with hematoxylin and eosin (H&E) staining of each group. The hepatorrhagia is greatly severe and the modified histological activity index (HAI) scores are significant higher in groups pretreated with indole derivatives, which represent a severer tissue injury and hepatocytes damage compared with VeAL group (Figures 1B, C). Of note, indole derivatives (IAA, ILA, IPA) administration along does not exhibit significant toxicity or induce inflammation response to mice in our preliminary experiment (Supplementary Figures 1A, B) and according to other studies (Ji et al., 2019; Xiao et al., 2020). Thus, results of biochemical and histologic analysis indicated that mice in indole derivatives (IAA, ILA, IPA) can sensitize mice to D-GalN/LPS induced-ALF.

### IAA administration aggravated D-GalN/LPS induced-liver inflammation and apoptosis

**Figure 2:**
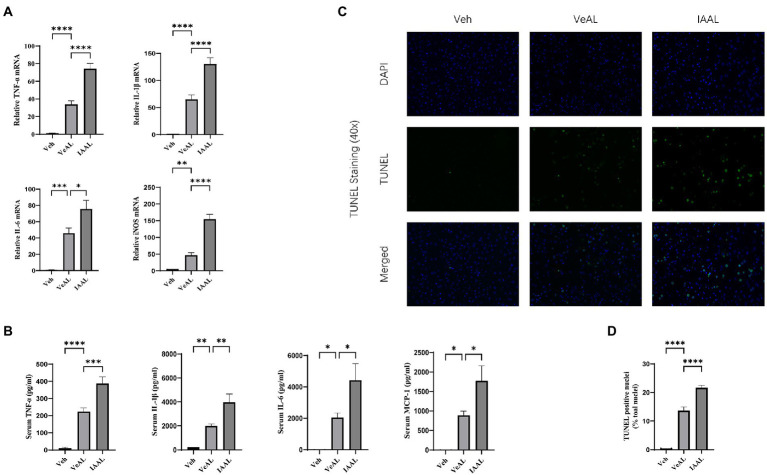
IAA administration aggravated D-GalN/LPS induced-Liver inflammation and apoptosis. **(A)** Relative expression of TNF-α, IL-1β, IL-6, and iNOS mRNA in liver. **(B)** Serum concentration of TNF-α, IL-1β, IL-6 and MCP-1. **(C)** Representative liver DAPI/TUNEL immunofluorescence staining (original magnification × 40, scale bar = 20 μm). **(D)** Percentage of TUNEL-positive cells in each group. The data are presented as the mean ± SEM. *p < 0.05; **p < 0.01; ***p < 0.001 and ****p < 0.0001 for the comparison. Veh, Vehicle (saline control); VeAL, Vehicle + D-GalN/LPS; IAAL, IAA + D-GalN/LPS. *n* = 10 in each group.

In order to get a clearer view of the role of the indole derivatives in the D-GalN/LPS induced-ALF, we chose the IAAL group for further investigation. TNF-α signaling is recognized as the crucial pathogenic and inflammatory factor in D-GalN/LPS induced-ALF (Maes et al., 2016). Thus, we evaluated the expression of TNF-α in liver with RT-qPCR. The relative expression of TNF-α, IL-1β, IL-6, and iNOS mRNA was significantly elevated in IAAL groups compared with VeAL group (Figure 2A). The quantification of serum cytokines TNF-α, IL-1β, IL-6, macrophage recruitment monocyte chemotactic protein-1 (MCP-1) were in consistence with RT-qPCR results (Figure 2B). We also evaluate the degree of hepatocytes apoptosis with terminal deoxynucleotidyl transferase-mediated dUTP nick end labeling (TUNEL) assay. The pretreatment of IAA significantly aggravated cell apoptosis in liver tissue (Figures 2C, D). Taken together, IAA can aggravate D-GalN/LPS induced-liver inflammation and apoptosis.

### IAA alters liver transcriptional expression in D-GalN/LPS induced-ALF

**Figure 3:**
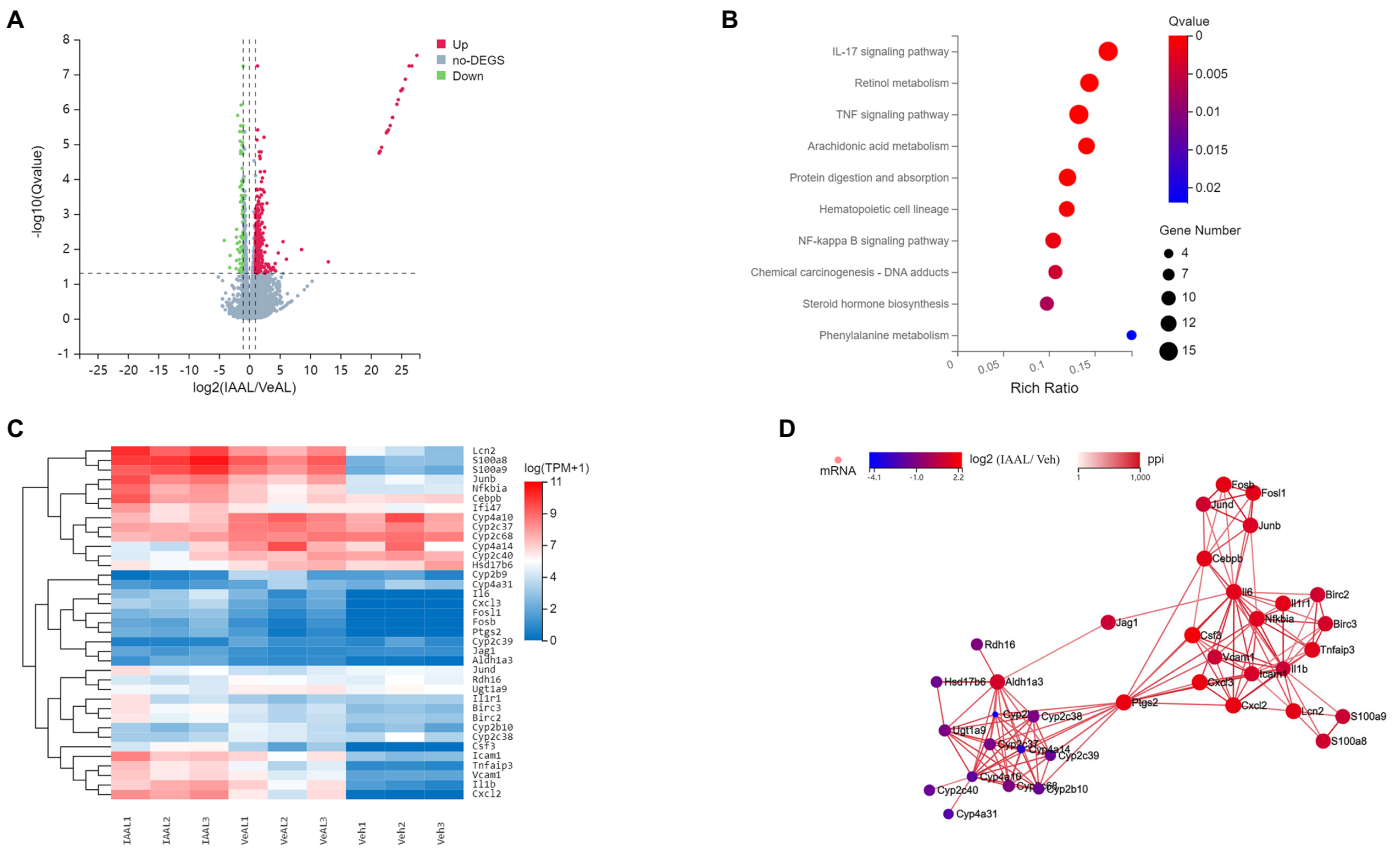
IAA alters liver transcriptome expression in D-GalN/LPS induced-ALF. **(A)** Volcano map for differential genes between VeAL and IAAL. **(B)** KEGG enrichment analyses of DEGs between VeAL and IAAL. **(C)** Heatmap of the DEGs from the four selected KEGG pathways. **(D)** Visualization of the four pathways’ network. Veh, Vehicle (saline control); VeAL, Vehicle + D-GalN/LPS; IAAL, IAA + D-GalN/LPS. *n* = 3 in each group.

The aforementioned data already demonstrate the pathogenic function of IAA administration in D-GalN/LPS-induced ALF, however the underlying signaling route remains unknown. To investigate the potential mechanism by which indole derivatives sensitize mice to D-GalN/LPS-induced ALF, an RNA sequence analysis was done to identify potential routes. 419 genes were identified as differentially expressed genes (DEGs) in the comparison between the IAAL and the VeAL using the criterion *q* ≤ 0.05 and|log2 (fold change)| ≥ 1. 350 DEGs were considerably elevated, whereas 69 DEGs were significantly downregulated (Figure 3A and Supplementary Figure 2A). Using the criteria Q 0.05, KEGG enrichment analysis of DEGs indicated that treatment of IAA substantially altered 28 pathways in VeAL mice. Inflammation, signal transduction, and metabolism made up the majority of the top 10 impacted pathways (Figure 3B). Four routes, including TNF signaling, NF-κB signaling, IL-17 signaling, and retinol metabolism pathway, were identified based on their regular fluctuation tendency, which demonstrated consistency with the progression of liver damage (Figure 3C). By mapping genes from these four major pathways, a protein–protein interaction (PPI) network was built to identify genes involved in ALI aggravation (Figure 3D). These findings showed that the aggravation of the condition might be ascribed to the control of transcriptional expression associated with inflammation, signal transduction, and metabolism.

### IAA enhanced the TNF-α/NF-κB signaling pathway with the up-regulation of Tlr2 instead of Tlr4 signaling in D-GalN/LPS induced-ALF

**Figure 4:**
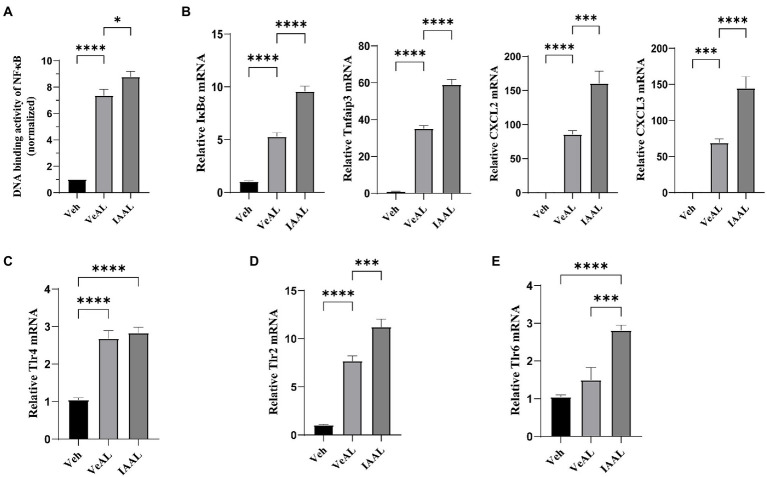
IAA enhanced the TNF-α/NF-κB signaling pathway with the up-regulation of Tlr2 instead of Tlr4 signaling in D-GalN/LPS induced-ALF. **(A)** The transcriptive activity of NF-κB (P65). **(B)** Relative expression of IκBα, Tnfaip3, Cxcl2 and Cxcl3 mRNA in liver. **(C–E)** Relative expression of Tlr4, Tlr2, Tlr6 mRNA in liver. The data are presented as the mean ± SEM. *p < 0.05; **p < 0.01; ***p < 0.001 and ****p < 0.0001 for the comparison. Veh, Vehicle (saline control); VeAL, Vehicle + D-GalN/LPS; IAAL, IAA + D-GalN/LPS. *n* = 10 in each group.

The results of RNA sequence analysis indicate the possible involvement of TNF signaling, NF-κB signaling, IL-17 signaling pathway in the aggravation of ALI. We first examined the expression levels of IL-17A in the liver. RT-qPCR showed no significant difference in IL-17A expression and serum cytokines assay only indicated a slight increase (about two folds compared with Veh) in IAAL (Supplementary Figure 3). Thus, the IL-17 signaling pathway is unlikely to play an influential role in our study. We next focused on the expression of TNF-α/NF-κB signaling pathway, which is a potent driver for inflammation response. We detect and find a significant elevation in the DNA-binding activity of NF-κB (P65) in IAAL, which is in consistent with our transcriptome results (Figure 4A). Additionally, IAA significantly elevated the expression of several crucial genes in TNF-α/NF-κB signaling including NFkBia and Tnfaip3 and downstream chemokines Cxcl2 and Cxcl3 (Figure 4B). The serum quantification of important downstream product IL-1β, IL-6, and TNF-α (Figure 2B) were significantly increased as above mentioned.

Toll-like receptor 4 (Tlr4)/NF-κB signaling is recognized as the primary pathogenic pathway in D-GalN/LPS induced-ALF activated by LPS administration (Maes et al., 2016). Thus, we hypothesized that IAA pretreatment may promote Tlr4 signaling. However, although D-GalN/LPS treatment significantly enhanced the gene expression of Tlr4 in liver compared with Veh, IAA pretreatment did not significantly alter it (Figure 4C). Nevertheless, we found that another vital toll-like receptor which can promote TNF-α/NF-κB pathway (Piao et al., 2016; Khan et al., 2021), toll-like receptor 2 (Tlr2), was significantly increased in IAAL compared with VeAL (Figure 4D). In addition, we also found that one of the essential co-factors of Tlr2 function, Toll-like receptor 6 (Tlr6; Ozinsky et al., 2000; Janeway and Medzhitov, 2002), exhibit significant increase in IAAL compared with VeAL (Figure 4E), which corroborates the activation of Tlr2 signaling. Taken together, IAA pretreatment may specifically up-regulate Tlr2/NF-κB signaling pathway under the challenge of D-GalN/LPS.

### IAA altered the composition of gut microbiota and induced dysbiosis of ileum microbiota in D-GalN/LPS induced-ALF

Tlr2 is an important sensor which can engage with bacterial components in gram-positive bacteria (Oliveira-Nascimento et al., 2012). On the other hand, IAA was reported to have the ability to influence microbial ecology (Shen et al., 2022). From this, we performed 16S RNA analyses in both colon and ileum contents to identify possible microbial involvement.

In colon, IAA pretreatment induces a significant shift of the composition of gut microbiota according to the unweighted UniFrac principal component analysis (PCA) analysis (Supplementary Figure 4A). The most altered taxonomic abundance of the microbiome was presented (Supplementary Figure 4B). However, we did not identify meaningful microbiome changes that could be responsible for Tlr2 activation.

**Figure 5:**
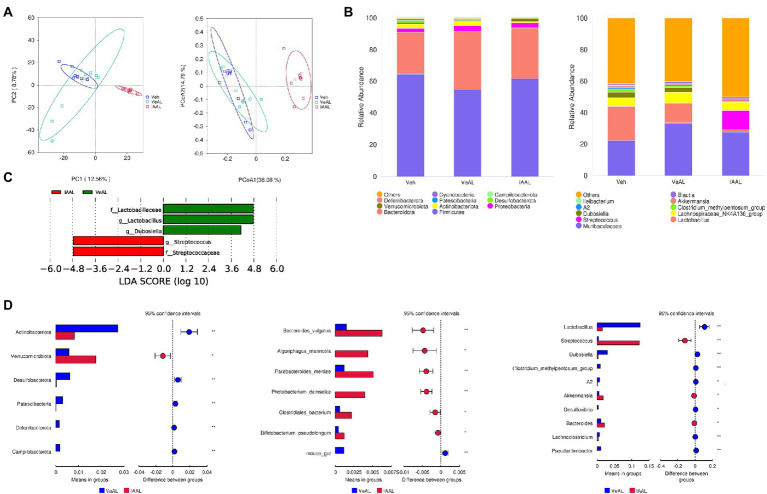
IAA altered the composition of gut microbiota and induced dysbiosis of ileum microbiota in D-GalN/LPS Induced-ALF. **(A)** Unweighted UniFrac PCoA and PCA plots. **(B)** Relative abundance of the 10 most abundant taxa at the phylum and genus levels. **(C)** LDA effect size plots. **(D)** *T*-tests between VeAL and IAAL in phylum, genus and species levels. *p < 0.05; **p < 0.01; ***p < 0.001 and ****p < 0.0001 for the comparison. Veh, Vehicle (saline control); VeAL, Vehicle + D-GalN/LPS; IAAL, IAA + D-GalN/LPS. *n* = 10 in each group.

As a note, we found encouraging results in the ileum microbiome, which may explain the activation of Tlr2 in the liver. The unweighted UniFrac principal coordinates analysis (PCoA) plot and principal component analysis (PCA) analysis also showed a significant shift in the composition of gut microbiota in IAAL (Figure 5A). The taxonomic abundance of the 10 most abundant microbiome was presented with phylum, family and genus levels (Figure 5B). Specifically, we found that IAA significantly increased the relative abundance of *Streptococcus* and reduced that of *Lactobacillus* (Figures 5C, D). The enrichment of gram-positive *Streptococcus* with underlying pathogenesis and the reduction of beneficial *Lactobacillus* indicates microbial dysbiosis in the ileum and may contribute to the up-regulation of Tlr2 signaling with IAA pretreatment.

### Alteration of certain microbial species in ileum dysbiosis was correlated with liver injury in D-GalN/LPS induced-ALF

**Figure 6:**
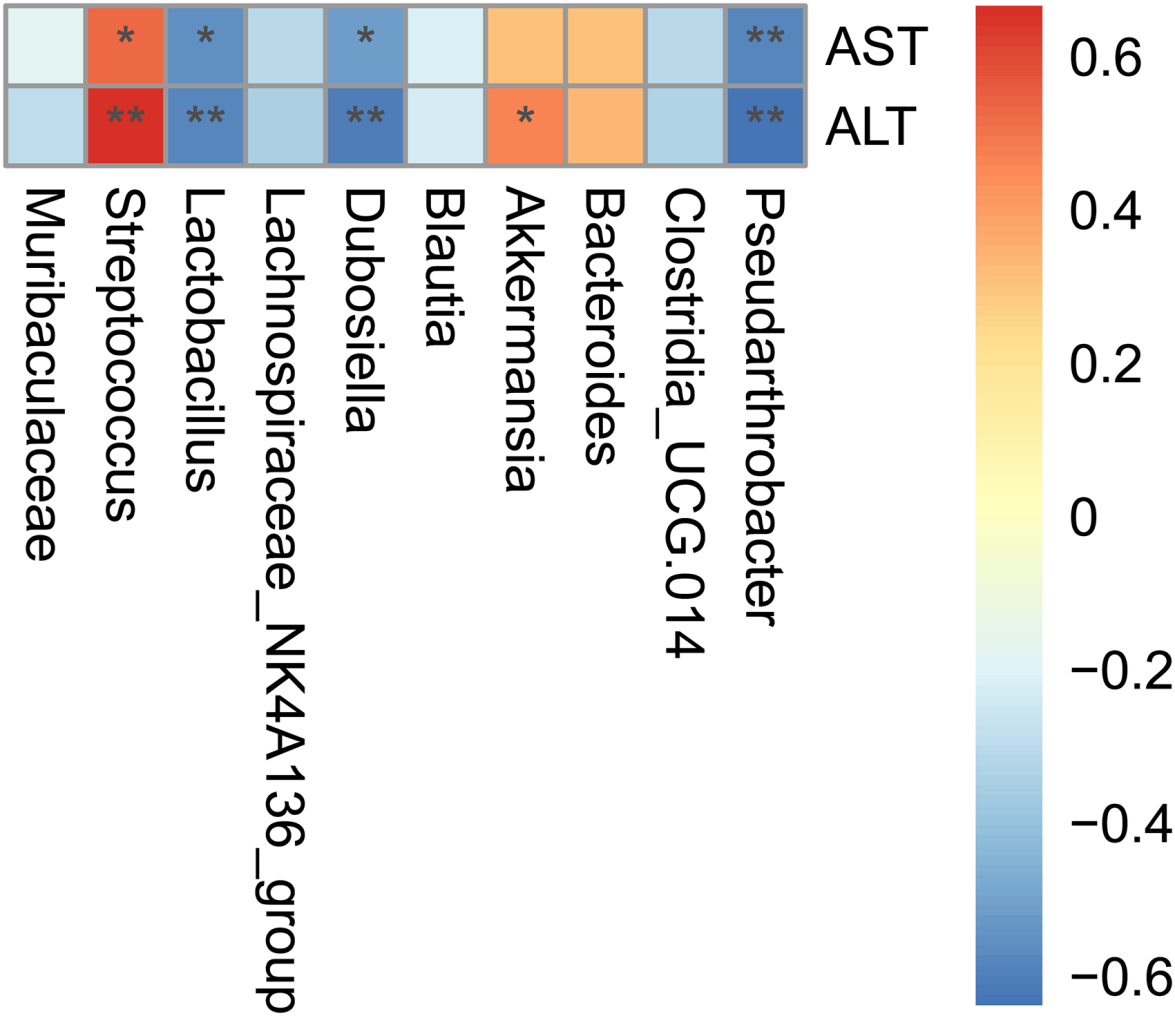
Alteration of certain microbial species in ileac dysbiosis was correlated with liver injury in D-GalN/LPS induced-ALF. Correlation analysis of differentially 10 most abundant microbial genera and liver aminotransferase. Correlation analysis was conducted by the Spearman rank correlation test, and red (positive correlation) and blue (negative correlation) colors represent different correlation coefficients. *p < 0.05; **p < 0.01; ***p < 0.001 and ****p < 0.0001 for the comparison. Veh, Vehicle (saline control); VeAL, Vehicle + D-GalN/LPS; IAAL, IAA + D-GalN/LPS. *n* = 10 in each group.

Spearman analysis was performed to identify the underlying correlation between microbial alteration and liver injury. Ten of the richest genera were presented. In consistency with our above findings, the relative abundance of *Streptococcus* exhibited positive correlation with ALT (*r* = 0.67)/AST (*r* = 0.53) levels and the relative abundance of *Lactobacillus* exhibited negative correlation with ALT (*r* = −0.55)/AST (*r* = −0.58) levels (Figure 6). Our findings suggested that the increased *Streptococcus* and reduced *Lactobacillus* may at least, partly be responsible for the up-regulation of Tlr2/NF-κB signaling pathway.

### Oral gavage of IAA activated liver AHR signaling and the activation of AHR signaling had no impact on D-GalN/LPS induced-ALF

**Figure 7:**
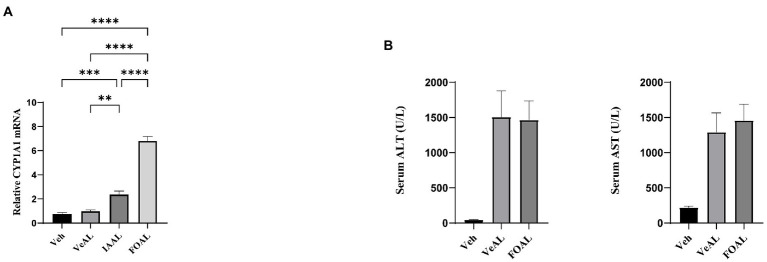
Oral gavage of IAA activated liver AHR signaling and the activation of AHR signaling had no impact on D-GalN/LPS induced-ALI. **(A)** Relative expression of CYP1A1 mRNA in liver. **(B)** Serum levels of ALT and AST. The data are presented as the mean ± SEM. **p < 0.01; ***p < 0.001 and ****p < 0.0001 for the comparison. Veh, Vehicle (saline control); VeAL, Vehicle + D-GalN/LPS; IAAL, IAA + D-GalN/LPS; FiAL, Ficz+ D-GalN/LPS. *n* = 10 in each group.

We also noticed that previous reports have revealed indole derivatives as important endogenous aryl hydrocarbon receptor (AHR) agonists and the activation of AHR signaling can aggravate APAP induced-ALF (Rothhammer et al., 2016; Schuran et al., 2021; Hezaveh et al., 2022). Thus, we hypothesize that the oral administration of IAA may aggravate the ALI through the activation of AHR signaling. However, we found that though oral administration of IAA and AHR agonist FICZ significantly increase the gene expression of CYP1A1 in liver (Figure 7A), FICZ along induced no significant difference in liver aminotransferase (Figure 7B). As a consequence, the activation of liver AHR signaling has no impact on D-GalN/LPS induced-ALF in our study.

## Discussion

Microbiome metabolites have been recognized as an important mediator connecting gut microbiota and host physiology (Caussy and Loomba, 2018; Canfora et al., 2019). Verification of the role of microbial metabolites in various diseases can help us better predict and comprehend how commensal species may influence the progression of various pathogens. Previous research strongly suggests that microbial metabolites, such as indole derivatives, have beneficial effects. In diseases related to the gut-liver axis, direct administration or indirect regulation of indole derivatives has been shown to be beneficial for anti-inflammatory and eubiosis maintenance (Agus et al., 2018; Ji et al., 2019; Zhao et al., 2019; Ma et al., 2020). Moreover, a number of studies have suggested the protective role of probiotics, which can inhibit endotoxin translocation in experimental ALF, but the role of microbial metabolism remains unknown (Nakamoto et al., 2017; Gong et al., 2018; Schneider et al., 2021). We originally planned to investigate whether indole derivatives could help suppress this signaling *via* possible regulation of gut microbiome and endotoxin translocation. However, our study revealed that certain indole derivatives, particularly IAA pretreatment, can sensitize mice to D-GalN/LPS induced ALF through increased liver inflammation, hepatocyte apoptosis and oxidative stress. Further analysis indicated that IAA may aggravate the liver injury *via* the up-regulation of Tlr2/NF-κB signaling pathway which may be mediated by the dysbiosis in ileum microbiota.

The activation of TNF-α/NF-κB signaling is the primary pathogenic pathway in D-GalN/LPS induced-ALF. D-GalN administration can sensitize hepatocytes to LPS challenge and collectively lead to the excessive activation of the classical LPS/Tlr4/NF-κB signaling characterized by massive production of inflammatory agents including TNF-α, IL-1β, IL-6 (Maes et al., 2016; Wang et al., 2020). The elimination of gut-derived LPS can protect mice from D-GalN/LPS-induced ALF, according to a previous study (Zheng et al., 2022). Microbial regulation of LPS/Tlr4 signaling may serve as a potential intervention target. Nevertheless, our intervention with IAA unexpectedly up-regulate TNF-α/NF-κB signaling according to our evaluation of DNA-binding activity of NF-κB (P65). Moreover, further PCR results do not support the involvement of Tlr4 signaling with IAA administration. As a consequence, oral gavage of IAA can up-regulate TNF-α/NF-κB pathway independent of Tlr4/NF-κB signaling.

Toll-like receptors are crucial components in innate immunity (Janeway and Medzhitov, 2002). Tlr2/NF-κB pathway is also an important candidate to drive downstream inflammation (Oh et al., 2012; Wang M. et al., 2019). Unlike Tlr4 which specifically engages with LPS from gram-negative (G-) bacteria, Tlr2 can engage with specific antigen lipteichoic acid (LTA) or peptidoglycan (PGN) from G+ bacteria (Takeuchi et al., 1999; Oliveira-Nascimento et al., 2012). Intriguingly, despite the fact that regular 16S RNA detection in colon contents did not yield correlating evidence, we detected an enrichment of G+ pathogenic *Streptococcus* and a reduction in *Lactobacillus* in the ileum. Notably, different studies have identified Tlr2 signaling as the primary immune pathway responding to *Streptococcus* species in pathogenic conditions (Sun et al., 2017; Li et al., 2020; Horn et al., 2022), which are in support of our findings. In addition, *Lactobacillus* is a common component of probiotics and the reduced *Lactobacillus* is a common dysbiosis phenotype in different diseases (Cani et al., 2008; Cukrowska et al., 2021; McDonnell et al., 2021). IAA may induce dysbiosis of ileum microbiota characterized by increased G+ pathogen and decreased beneficial specie. This microbial alteration provides a reasonable explanation for Tlr2 activation in our study. However, simple impact of oral administration of IAA is not enough to induce pathogenic phenotype. Pathogenic stimulation from LPS is the primary cause Tlr2 activation (VeAL vs. Veh) and IAA treatment only contributes to a less increase of Tlr2 expression in our study (IAAL vs. VeAL) (Figure 4C). We assumed that IAA is not enough to pathogenically activate Tlr2 signaling without the co-effect of LPS stimulation. IAA-induced dysbiosis acts as a contributary instead of an initial factor. Taken together, IAA may sensitize D-GalN/LPS induced-liver injury *via* the up-regulation of Tlr2/NF-κB pathway.

In addition, several important studies have indicated that indole derivatives are important endogenous AHR agonists (Rothhammer et al., 2016; Hezaveh et al., 2022). AHR signaling plays an important but controversial role in the inflammation response (Schuran et al., 2021; Teixeira et al., 2022). Thus, we also examined the role of AHR signaling with AHR agonist FICZ. Results indicated no corresponding involvement of AHR in our study.

However, this study still leaves some points in vagueness. The role of IAA in our study is contradictory to previous findings (Ji et al., 2019; Shen et al., 2022). In our study, not only did IAA exhibit no help on the regulation of endotoxin translocation or liver inflammatory signaling, but also induced ileum dysbiosis. On the one hand, we hypothesize that short-term gavage of IAA (1 week) in our study is not enough to induce direct and equal influence of long-term injection of IAA (4 weeks) in systemic signaling or gut microbiota. On the other hand, though this study identified similar pattern in gut microbiota regulation with previous report such as increased *Bacteroides* in colon (Shen et al., 2022), the unique role of IAA in ileum microbiota homeostasis is firstly reported. The different route for administration (oral gavage vs. intraperitoneal injection) may exert distinct impact especially on the composition of gut microbiota.

This study has several limitations. Firstly, IAA may regulate microbial or host metabolism according to previous report and our transcriptomic analysis (Agus et al., 2018). The lack of additional detection of liver or serum metabolome in the present study may have prevented identification of the metabolic signaling pathway that contributes to disease progression. Secondly, even though we have confirmed the safety and tolerability of IAA gavage in our preliminary study, assays on the transcriptome and microbiome in simple IAA pretreatment may provide evidences to clarify our study further. Thirdly, the current conclusion is still a possible explanation, and additional testing with Tlr2 antagonists or gene-deficient animals is required to confirm our conclusion.

In conclusion, our study is the first to report the pathogenic effect of indole derivatives, specifically IAA, in D-GalN/LPS-induced-ALF, as well as the IAA-induced dysbiosis in ileum microbiota. In light of the fact that previous studies primarily identified indole derivatives as beneficial microbial metabolites associated with anti-inflammation and microbiota homeostasis (Wlodarska et al., 2017; Ma et al., 2020; Qayed et al., 2021; Shen et al., 2022), our findings indicate that indole derivatives, especially IAA and related commensal species in ALF need more caution. We also noticed that a previous study has suggested the pathogenic role of IPA in CCl4-induced liver injury (Liu F. et al., 2021). In addition, our study revealed that variations in the microbiota composition and microbial metabolism may influence individual susceptibility to ALF. Individual susceptibility induced by indole metabolism may be clarified through additional research that includes a comprehensive examination of indole derivatives and observation of clinical samples.

## Data availability statement

The datasets presented in this study can be found in online repositories. The names of the repository/repositories and accession number (s) can be found at: https://www.ncbi.nlm.nih.gov/, Sequence Read Archive (SRA) database under the BioProject accession code PRJNA909465.

## Ethics statement

The animal study was reviewed and approved by the Animal Experimental Ethical Inspection of the First Affiliated Hospital, Zhejiang University of Medicine.

## Author contributions

XP and ZZ: conceptualization, writing, and original draft preparation. XP, JL, ZL, YH, JC, and QW: experiments operation. BW and LL: writing, review, and editing. YY, ZZ, and XP: visualization. LL: supervision. All authors have read and agreed to the published version of the manuscript.

## Funding

This research was funded by the National Natural Science Foundation of China (81790631).

## Conflict of interest

The authors declare that the research was conducted in the absence of any commercial or financial relationships that could be construed as a potential conflict of interest.

## Publisher’s note

All claims expressed in this article are solely those of the authors and do not necessarily represent those of their affiliated organizations, or those of the publisher, the editors and the reviewers. Any product that may be evaluated in this article, or claim that may be made by its manufacturer, is not guaranteed or endorsed by the publisher.

## Footnotes

^1^ https://www.3dhistech.com/solutions/caseviewer/
