# Supplementary material for: Microbial metabolites indole derivatives sensitize mice to D-GalN/LPS induced-acute liver failure via the Tlr2/NF-κB pathway
Source: Front Microbiol. 2023 Jan 6;13:1103998. doi: 10.3389/fmicb.2022.1103998 (PMC9852867; doi:10.3389/fmicb.2022.1103998)
Supplement: Supplementary file 1 [file Data_Sheet_1.docx]

Supplementary Material

# Supplementary Data

# Statistical analysis

# Transcriptome analysis

# The sequencing data was filtered with SOAPnuke (v1.5.2) [1] by (1) Removing reads containing sequencing adapter; (2) Removing reads whose low-quality base ratio (base quality less than or equal to 5) is more than 20%; (3) Removing reads whose unknown base ('N' base) ratio is more than 5%, afterwards clean reads were obtained and stored in FASTQ format. The clean reads were mapped to the reference genome using HISAT2 (v2.0.4) [2]. After that, Ericscript (v0.5.5) [4] and rMATS (V3.2.5) [5] were used to fusion genes and differential splicing genes (DSGs), respectively. Bowtie2 (v2.2.5) [6] was applied to align the clean reads to the gene set, a database for this organism built by BGI (Beijing Genomic Institute in ShenZhen), which known and novel, coding transcripts were included, then expression level of gene was calculated by RSEM (v1.2.12) [7]. The heatmap was drawn by pheatmap (v1.0.8) [8] according to the gene expression in different samples. Essentially, differential expression analysis was performed using the DESeq2(v1.4.5) [9] with Q value ≤ 0.05. To take insight to the change of phenotype, GO (http://www.geneontology.org/) and KEGG (https://www.kegg.jp/) enrichment analysis of annotated different expression gene was performed by Phyper (https://en.wikipedia.org/wiki/Hypergeometric_distribution) based on Hypergeometric test. The significant levels of terms and pathways were corrected by Q value with a rigorous threshold (Q value ≤ 0.05) by Bonferroni.

# Bacteria 16s RNA

# Paired-end reads were assigned to samples based on their unique barcodes and were truncated by cutting off the barcodes and primer sequences. Paired-end reads were merged using FLASH (Version 1.2.11, http://ccb.jhu.edu/software/FLASH/) [10], a very fast and accurate analysis tool designed to merge paired-end reads when at least some of the reads overlap with the reads generated from the opposite end of the same DNA fragment, and the splicing sequences were called Raw Tags. Quality filtering on the raw tags were performed using the fastp (Version 0.20.0) software to obtain high-quality Clean Tags. The Clean Tags were compared with the reference database (Silva database https://www.arbsilva.de/ for 16S/18S, Unite database https://unite.ut.ee/ for ITS) using Vsearch (Version 2.15.0) to detect the chimera sequences, and then the chimera sequences were removed to obtain the Effective Tags [11]. For the Effective Tags obtained previously, denoise was performed with DADA2 or deblur module in the QIIME2 software (Version QIIME2-202006) to obtain initial ASVs (Amplicon Sequence Variants) (default: DADA2), and then ASVs with abundance less than 5 were filtered out [12]. Species annotation was performed using QIIME2 software. For 16S/18S, the annotation database is Silva Database, while for ITS, it is Unite Database. In order to study phylogenetic relationship of each ASV and the differences of the dominant species among different samples (groups), multiple sequence alignment was performed using QIIME2 software. The absolute abundance of ASVs was normalized using a standard of sequence number corresponding to the sample with the least sequences. Subsequent analysis of alpha diversity and beta diversity were all performed based on the output normalized data. Alpha diversity and beta diversity was calculated in QIIME2.

# Supplementary Figures and Tables

## Supplementary Figures

A


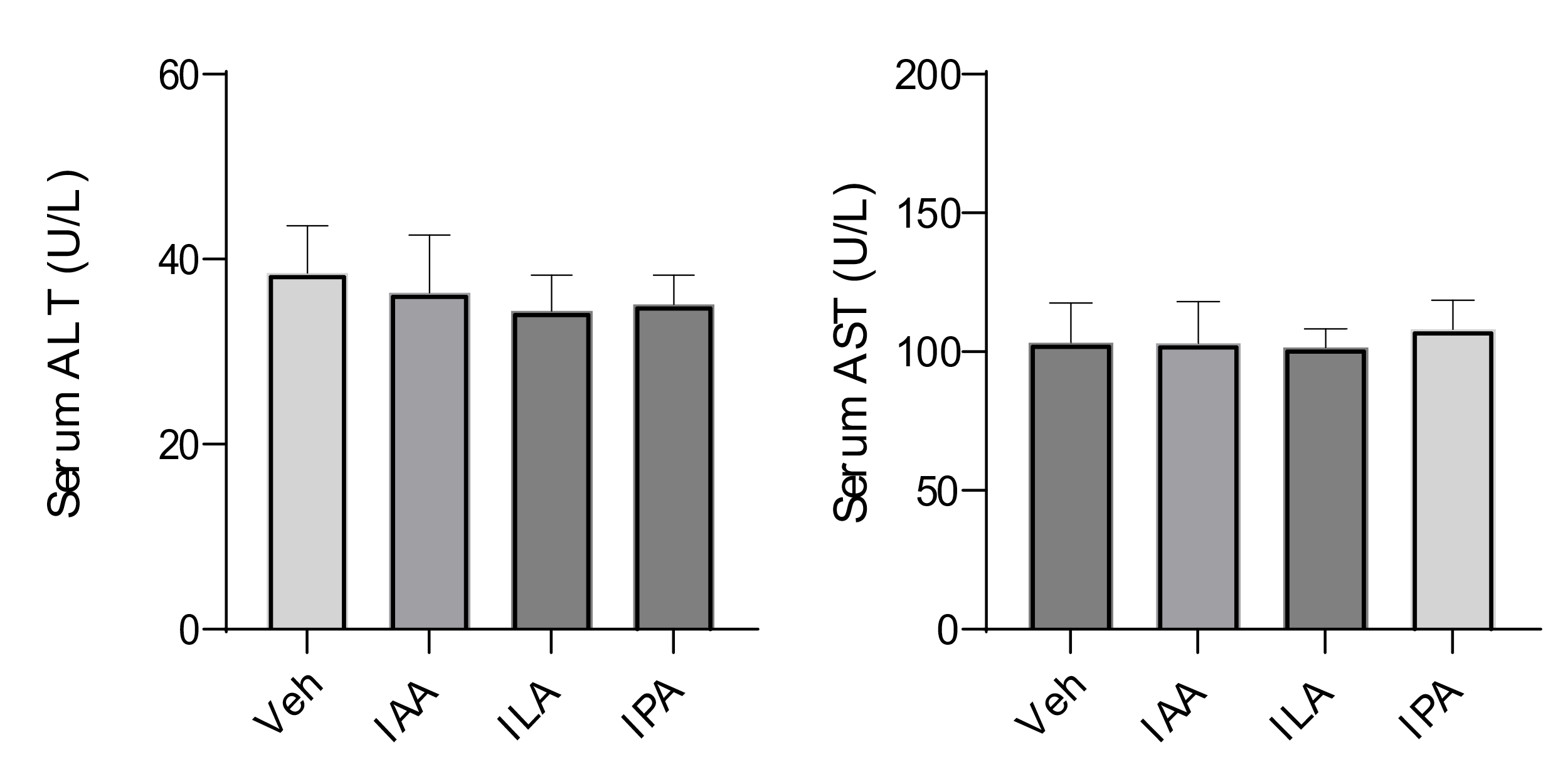


B


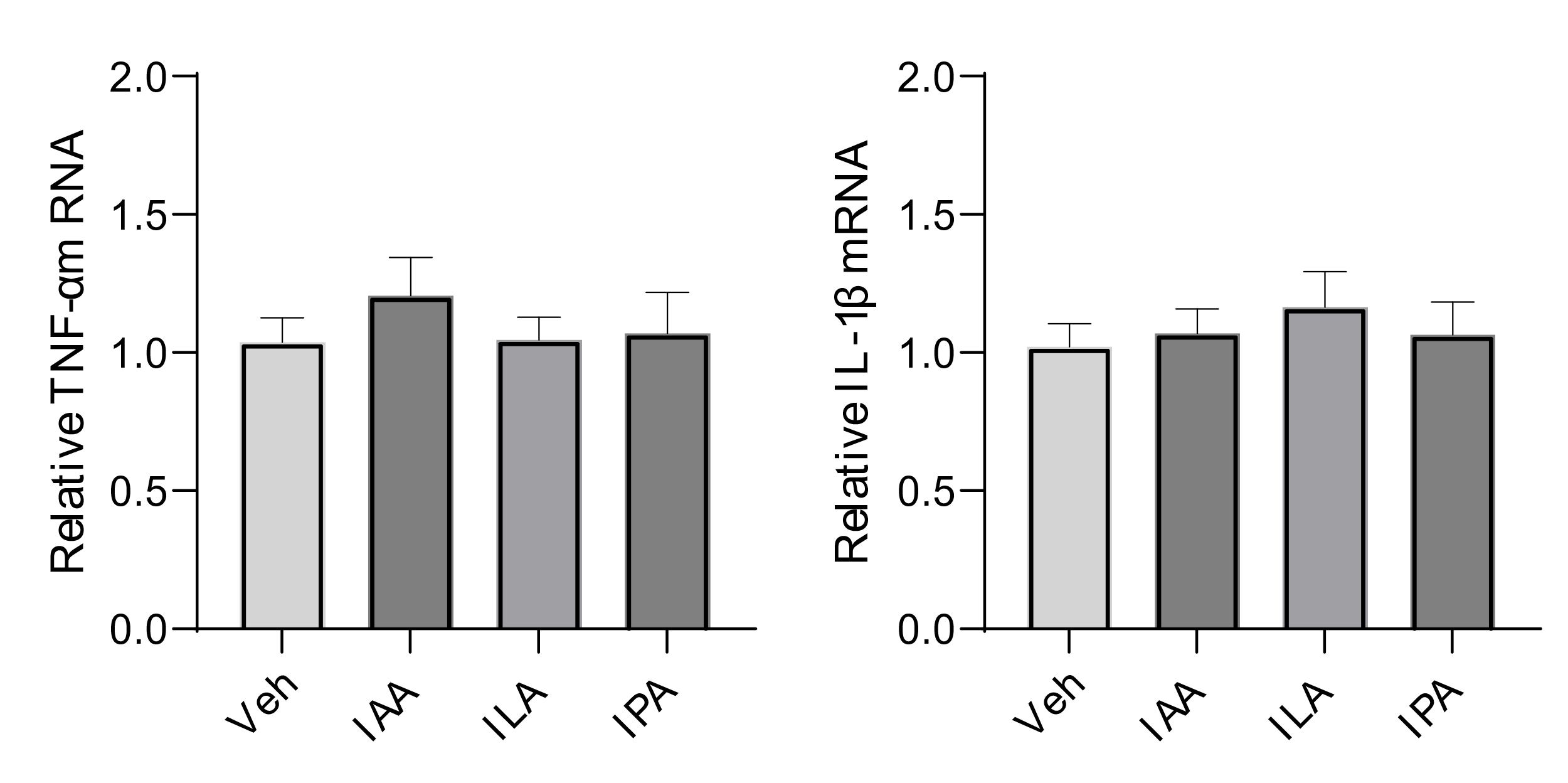


**Supplementary Figure 1.** Pretreatment of indole derivatives exhibited no toxicity in mice. (A) Serum levels of ALT and AST. (B) Relative expression of TNF-α, IL-1βmRNA in liver. The data are presented as the mean ± SEM. *, P < 0.05; **, P < 0.01; ***, P < 0.001 and ****, P < 0.0001 for the comparison. Veh, Vehicle (saline control); IAA, indole-3-acetic acid; ILA, indole-3-lactic acid; IPA, indolepropionic acid. n = 10 in each group.


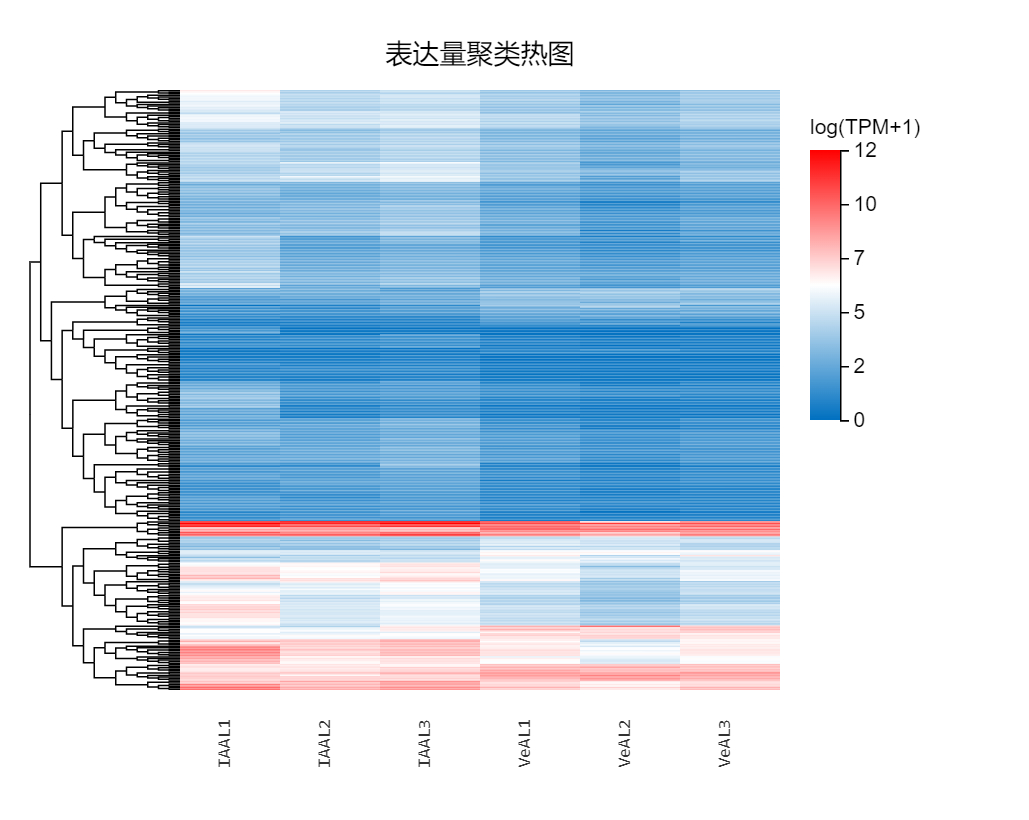


**Supplementary Figure 2.** Heatmap of all the DEGs between IAAL and VeAL.


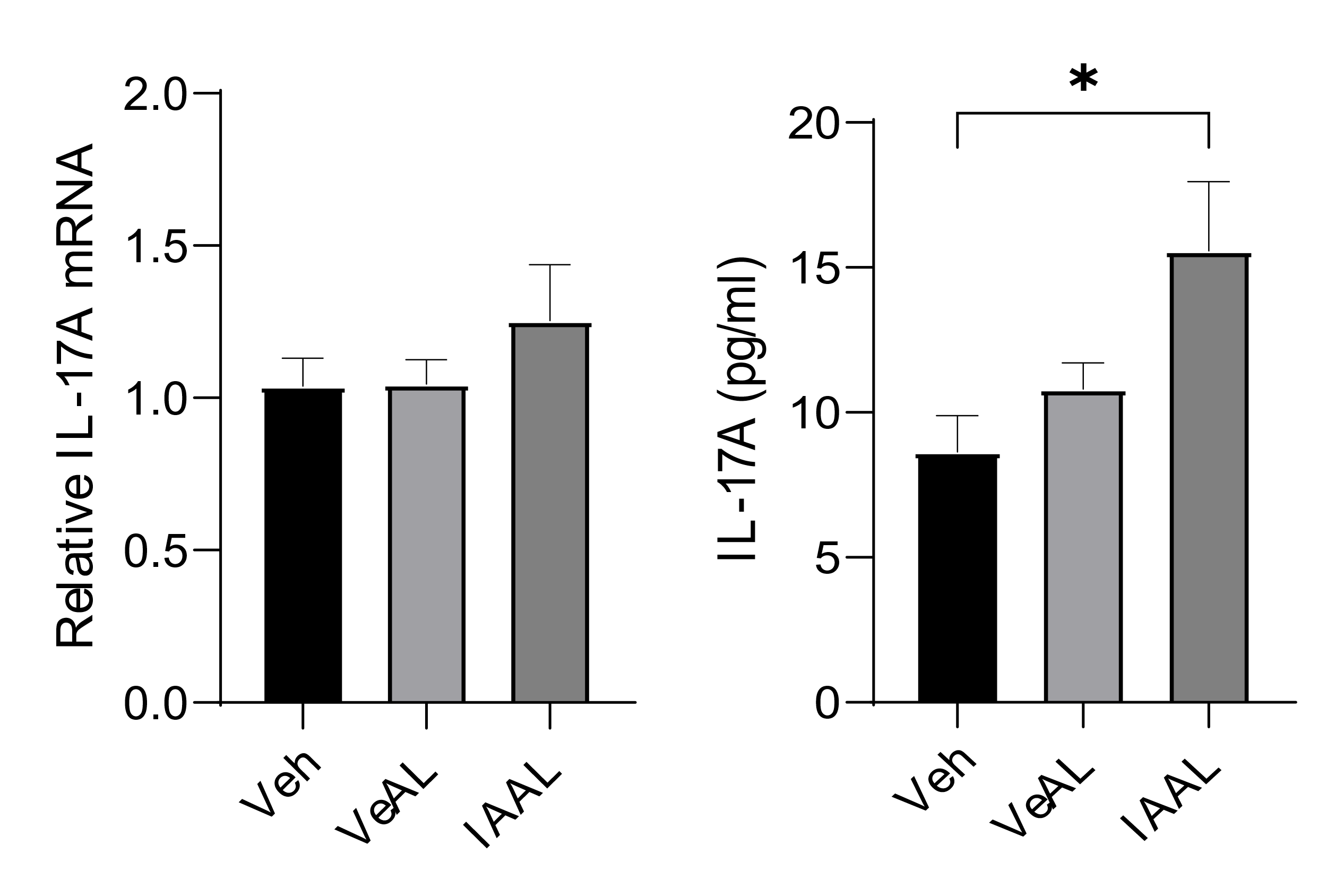


**Supplementary Figure 3.** Relative expression of IL-17A mRNA in liver and serum concentration of IL-17A. The data are presented as the mean ± SEM. *, P < 0.05; **, P < 0.01; ***, P < 0.001 and ****, P < 0.0001 for the comparison. Veh, Vehicle (saline control); VeAL, Vehicle + D-GalN/LPS; IAAL, IAA + D-GalN/LPS. n = 10 in each group.

A


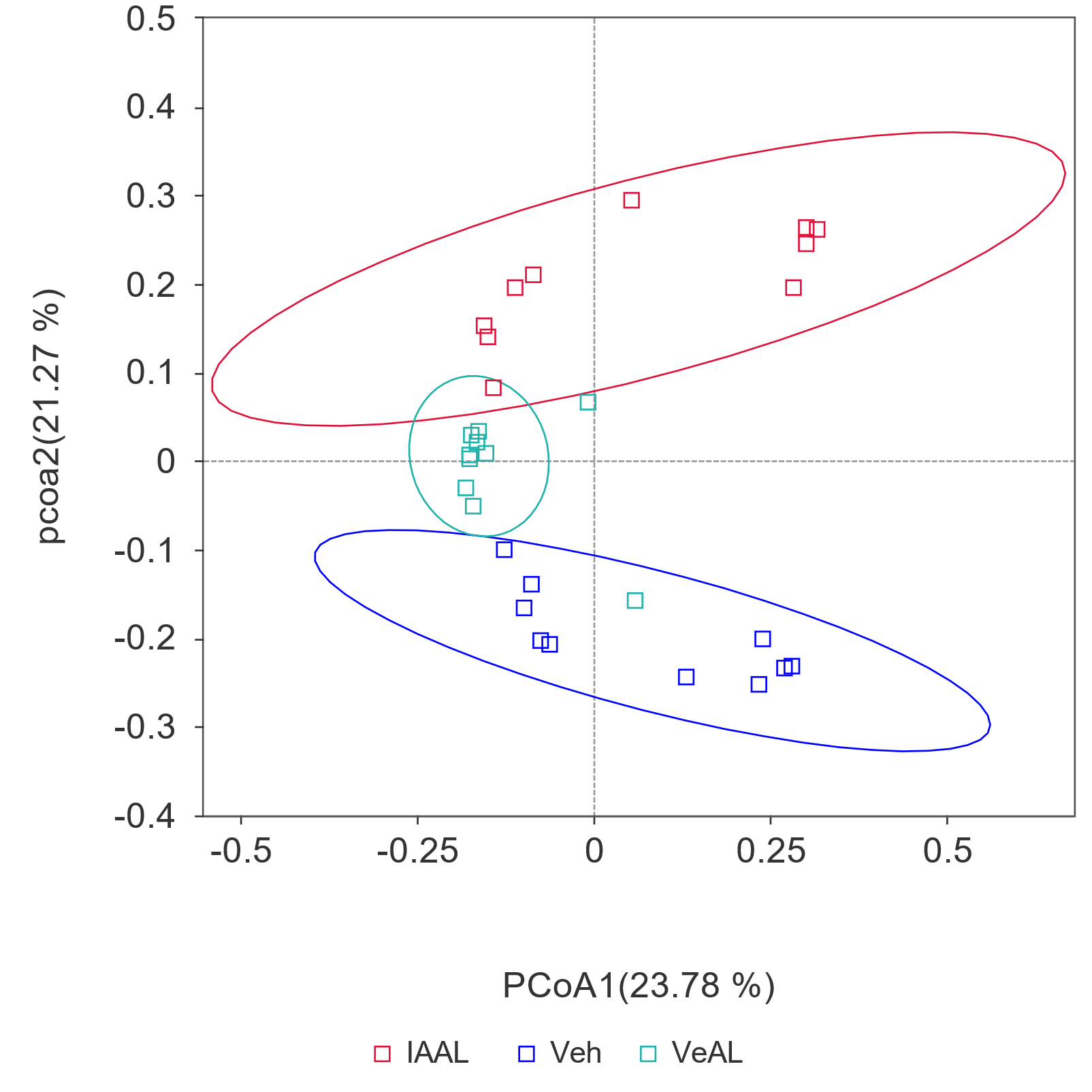


B


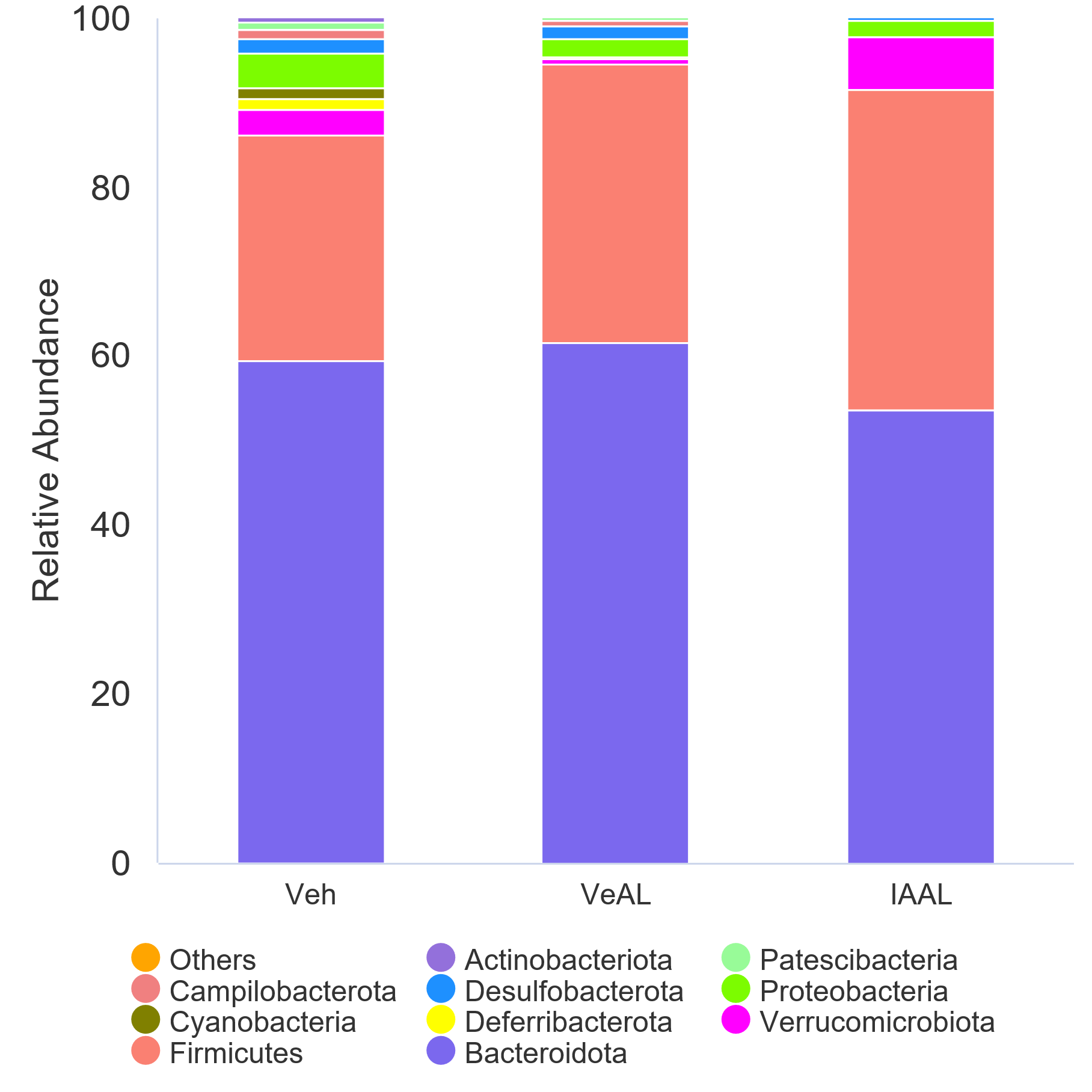

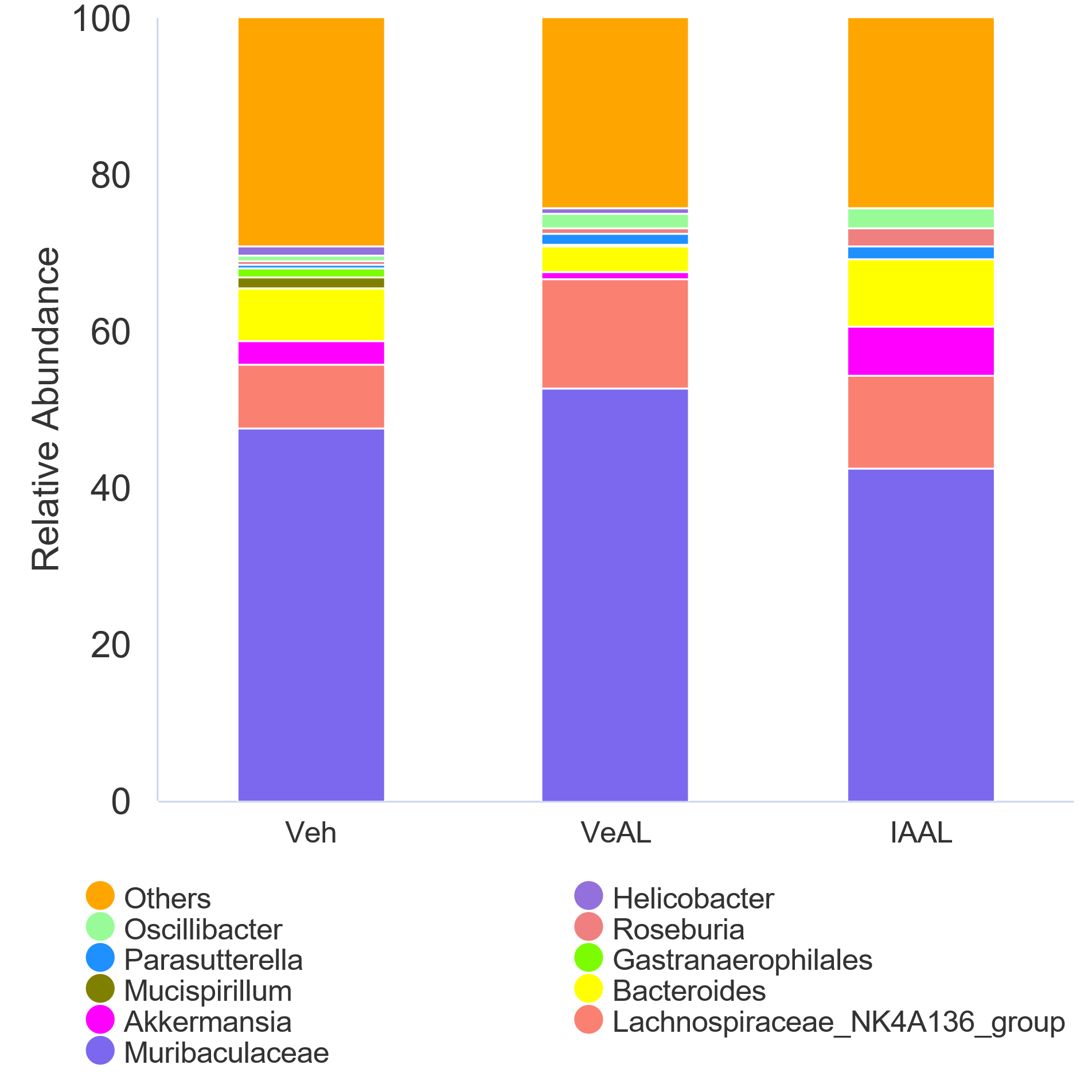


C


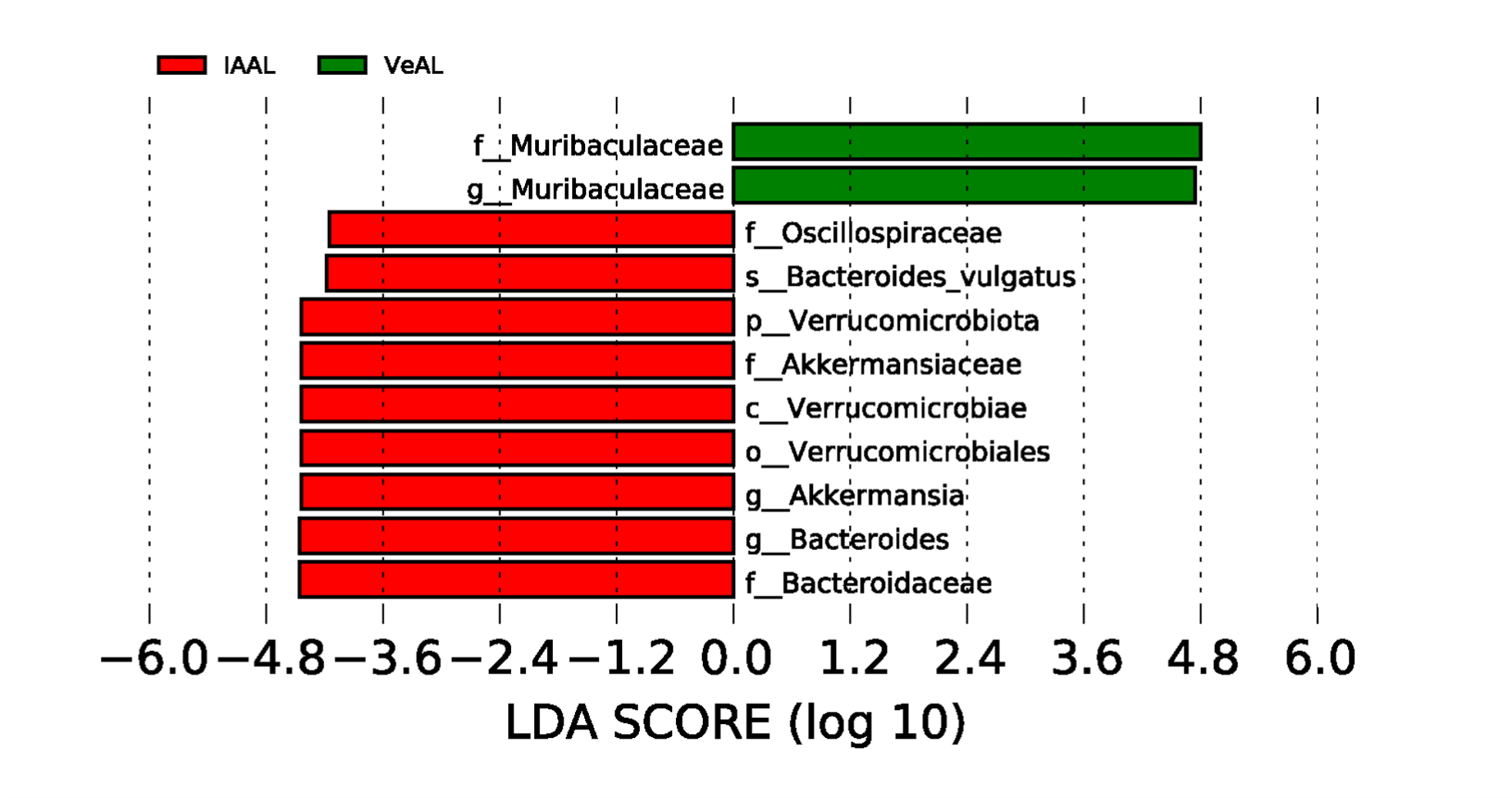


D


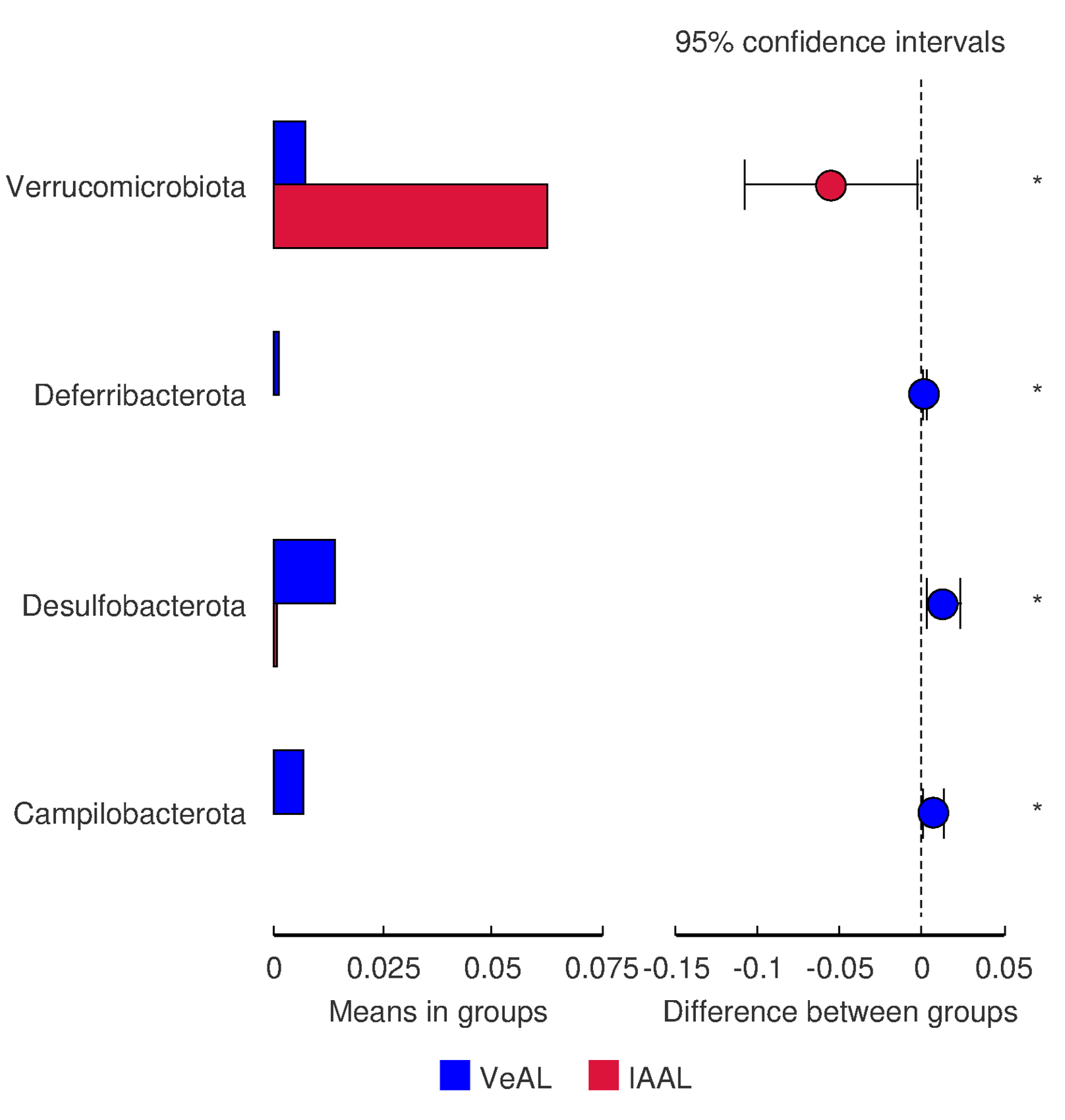

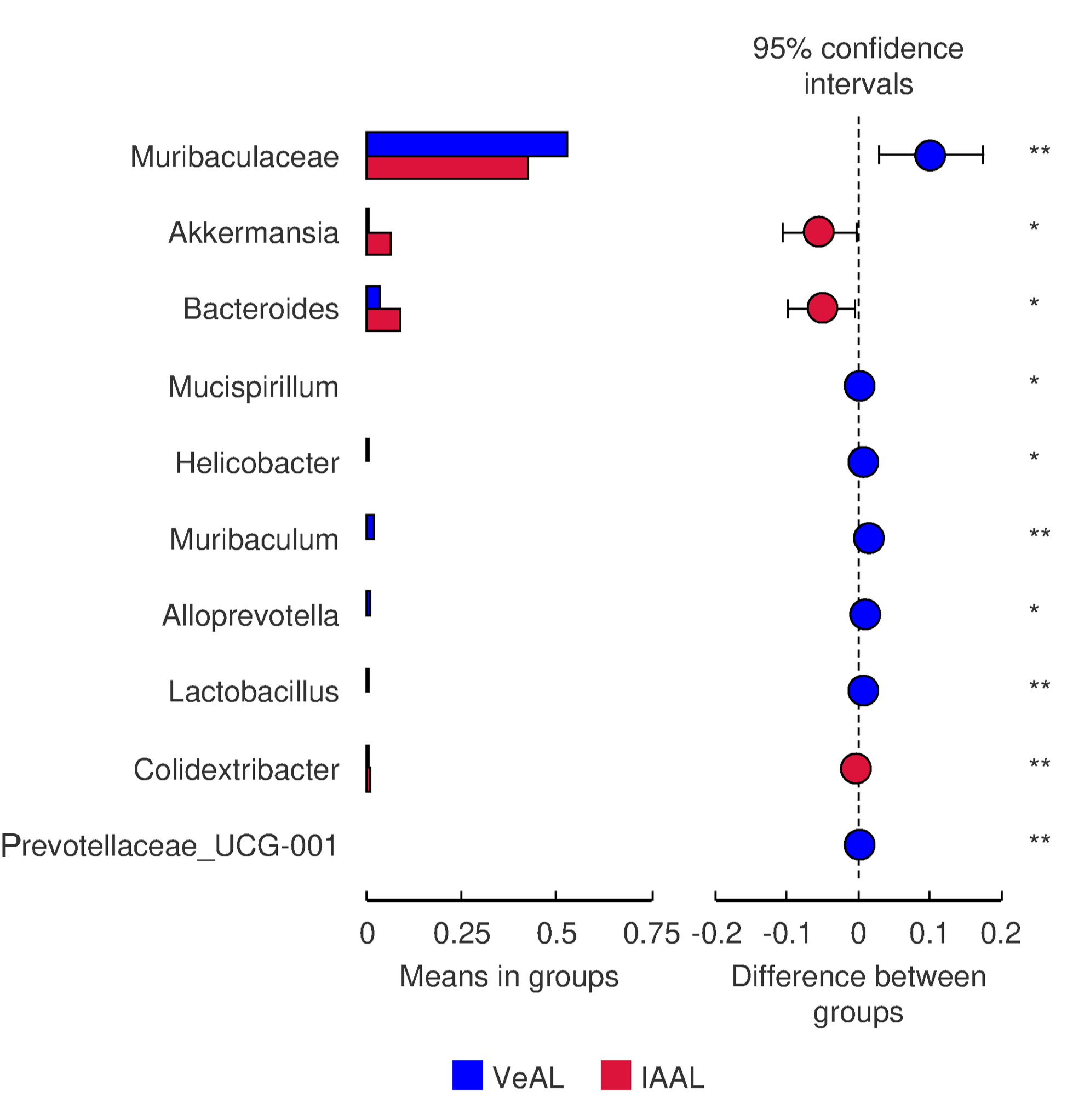


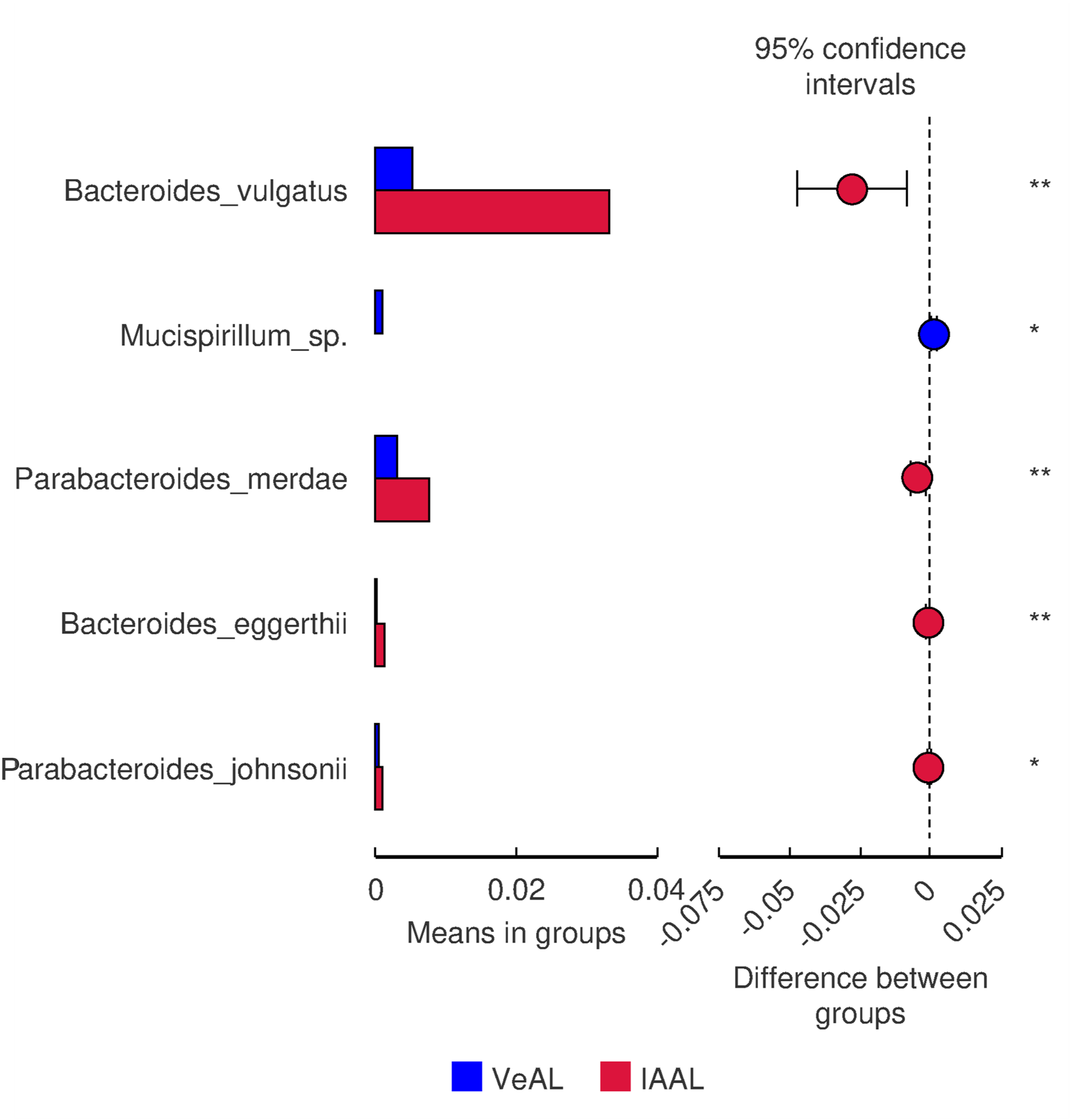


**Supplementary Figure 4.** IAA altered the composition of colon microbiota in D-GalN/LPS Induced-ALF. (A) Unweighted UniFrac PCoA plot. (B) Relative abundance of the ten most abundant taxa at the phylum and genus levels. (C) LDA effect size plot. (D) T-tests between VeAL and IAAL in phylum, genus and species levels. *, P < 0.05; **, P < 0.01; ***, P < 0.001 and ****, P < 0.0001 for the comparison. Veh, Vehicle (saline control); VeAL, Vehicle + D-GalN/LPS; IAAL, IAA + D-GalN/LPS. n = 10 in each group.

## Supplementary Table

**Supplementary Table 1** Primer sequence information for quantitative real-time PCR assay

| Target gene |  |
| --- | --- |
| Gapdh f | AGGTCGGTGTGAACGGATTTG |
| Gapdh r | TGTAGACCATGTAGTTGAGGTCA |
| il-1β f | GAAATGCCACCTTTTGACAGTG |
| il-1β r | TGGATGCTCTCATCAGGACAG |
| Tnf-α r | CAGGCGGTGCCTATGTCTC |
| Tnf-α f | CGATCACCCCGAAGTTCAGTAG |
| il-6 f | TAGTCCTTCCTACCCCAATTTCC |
| il-6 r | TTGGTCCTTAGCCACTCCTTC |
| il-17a f | TTTAACTCCCTTGGCGCAAAA |
| il-17a r | CTTTCCCTCCGCATTGACAC |
| iNOS f | GTTCTCAGCCCAACAATACAAGA |
| iNOS r | GTGGACGGGTCGATGTCAC |
| Cyp1a1 f | CAGTCCCTCCTTACAGCCCAA |
| Cyp1a1 r | GCCAGTAACCTCCCCAAACT |
| IκBα f | TGAAGGACGAGGAGTACGAGC |
| IκBα r | TTCGTGGATGATTGCCAAGTG |
| Tnfaip3 f | GAACAGCGATCAGGCCAGG |
| Tnfaip3 r | GGACAGTTGGGTGTCTCACATT |
| CXCL2 f | TTAAAAACCTGGATCGGAACCAA |
| CXCL2 r | GCATTAGCTTCAGATTTACGGGT |
| CXCL3 f | GAAAGGAGGAAGCCCCTCAC |
| CXCL3 r | TGGCCAGCCAAGGAATACTG |
| Tlr4 f | CGCTTTCACCTCTGCCTTCACTACAG |
| Tlr4 r | ACACTACCACAATAACCTTCCGGCTC |
| Tlr2 f | GCAAACGCTGTTCTGCTCAG |
| Tlr2 r | AGGCGTCTCCCTCTATTGTATT |
| Tlr6 f | TGAGCCAAGACAGAAAACCCA |
| Tlr6 r | GGGACATGAGTAAGGTTCCTGTT |

The primers sequences were acquired from Primer bank (<https://pga.mgh.harvard.edu/primerbank/>) or NCBI Primer-BLAST (<https://blast.ncbi.nlm.nih.gov/>)

References

[1] Li R, Li Y, Kristiansen K, Wang J. (2008). SOAP: short oligonucleotide alignment program. Bioinformatics. 24(5):713-4

[2] Kim, D., Langmead, B. & Salzberg, S. L. HISAT: a fast spliced aligner with low memory requirements. Nat. Methods 12, 357-360 (2015).

[3] Matteo Benelli, Chiara Pescucci, Giuseppina Marseglia, Marco Severgnini, Francesca Torricelli, Alberto Magi, Discovering chimeric transcripts in paired-end RNA-seq data by using EricScript, Bioinformatics, Volume 28, Issue 24, December 2012, Pages 3232–3239.

[4] Shen, S. et al. rMATS: Robust and flexible detection of differential alternative splicing fromreplicate RNA-Seq data. Proc. Natl Acad. Sci. USA 111, E5593-E5601 (2014).

[5] Langmead, B. et al. Fast gapped-read alignment with Bowtie 2. Nat. Methods 9, 357-359(2012).

[6] Li, B. & Dewey, C. N. RSEM: accurate transcript quantification from RNA-Seq data with or without a reference genome. BMC Bioinformatics 12, 323 (2011).

[7] Raivo Kolde. Package ‘pheatmap’. 2019-01-04 13:50:12 UTC.

[8] Love, M. I., Huber, W. & Anders, S. Moderated estimation of fold change and dispersion for RNA-seq data with DESeq2. Genome Biol. 15, 550 (2014).

[9] Abdi, H. The Bonferonni and Sˇidák Corrections for Multiple Comparisons. Encycl Meas Stat. 2007; 1: 1–9.

[10] Magoč T, Salzberg S L. FLASH: fast length adjustment of short reads to improve genome assemblies. Bioinformatics 27.21 (2011): 2957-2963.

[11] Haas, Brian J., et al. Chimeric 16S rRNA sequence formation and detection in Sanger and 454-pyrosequenced PCR amplicons.Genome research 21.3 (2011): 494-504.

[12] Li Minjuan,Shao Dantong,Zhou Jiachen et al. Signatures within esophageal microbiota with progression of esophageal squamous cell carcinoma.[J] .Chin J Cancer Res, 2020, 32: 755-767.
